# Reduced polymorphism of *Plasmodium vivax* early transcribed membrane protein (PvETRAMP) 11.2

**DOI:** 10.1186/s13071-023-05851-9

**Published:** 2023-07-17

**Authors:** Edvige Perrotti, Mariangela L’Episcopia, Michela Menegon, Irene S. Soares, Angel Rosas-Aguirre, Niko Speybroeck, Alejandro LLanos-Cuentas, Didier Menard, Marcelo Urbano Ferreira, Carlo Severini

**Affiliations:** 1 Department of Infectious Diseases, Istituto Superiore Di Sanità, Rome, Italy; 2 Department of Clinical and Toxicological Analyses, School of Pharmaceutical Sciences, University of São Paulo, São Paulo, Brazil; 3 Research Institute of Health and Society (IRSS), Université Catholique de Louvain, Brussels, Belgium; 4 Instituto de Medicina Tropical “Alexander Von Humboldt”, Universidad Peruana Cayetano Heredia, Lima, Peru; 5 Laboratoire de Parasitologie Et Mycologie Médicale, Les Hôpitaux Universitaires de Strasbourg, Strasbourg, France; 6 Institut de Parasitologie Et Pathologie Tropicale, Université de Strasbourg, Strasbourg, France; 7 Malaria Genetics and Resistance Unit–INSERM U1201, Institut Pasteur, Paris, France; 8 Department of Parasitology, Institute of Biomedical Sciences, University of São Paulo, São Paulo, Brazil; 9 Global Health and Tropical Medicine, Instituto de Higiene e Medicina Tropical, Universidade NOVA de Lisboa, Lisbon, Portugal

**Keywords:** PvETRAMP11.2, *Plasmodium vivax*, Genetic variation, Polymorphism, Antigen

## Abstract

**Background:**

ETRAMP11.2 (PVX_003565) is a well-characterized protein with antigenic potential. It is considered to be a serological marker for diagnostic tools, and it has been suggested as a potential vaccine candidate. Despite its immunological relevance, the polymorphism of the *P. vivax* ETRAMP11.2 gene (*pvetramp11.2*) remains undefined. The genetic variability of an antigen may limit the effectiveness of its application as a serological surveillance tool and in vaccine development and, therefore, the aim of this study was to investigate the genetic diversity of *pvetramp11.2* in parasite populations from Amazonian regions and worldwide. We also evaluated amino acid polymorphism on predicted B-cell epitopes. The low variability of the sequence encoding PvETRAMP11.2 protein suggests that it would be a suitable marker in prospective serodiagnostic assays for surveillance strategies or in vaccine design against *P. vivax* malaria.

**Methods:**

The *pvetramp11.2* of *P. vivax* isolates collected from Brazil (*n* = 68) and Peru (*n* = 36) were sequenced and analyzed to assess nucleotide polymorphisms, allele distributions, population differentiation, genetic diversity and signature of selection. In addition, sequences (*n* = 104) of seven populations from different geographical regions were retrieved from the PlasmoDB database and included in the analysis to study the worldwide allele distribution. Potential linear B-cell epitopes and their polymorphisms were also explored.

**Results:**

The multiple alignments of 208 *pvetramp11.2* sequences revealed a low polymorphism and a marked geographical variation in allele diversity. Seven polymorphic sites and 11 alleles were identified. All of the alleles were detected in isolates from the Latin American region and five alleles were detected in isolates from the Southeast Asia/Papua New Guinea (SEA/PNG) region. Three alleles were shared by all Latin American populations (H1, H6 and H7). The H1 allele (reference allele from Salvador-1 strain), which was absent in the SEA/PNG populations, was the most represented allele in populations from Brazil (54%) and was also detected at high frequencies in populations from all other Latin America countries (range: 13.0% to 33.3%). The H2 allele was the major allele in SEA/PNG populations, but was poorly represented in Latin America populations (only in Brazil: 7.3%). *Plasmodium vivax* populations from Latin America showed a marked inter-population genetic differentiation (fixation index [Fst]) in contrast to SEA/PNG populations. Codon bias measures (effective number of codons [ENC] and Codon bias index [CBI]) indicated preferential use of synonymous codons, suggesting selective pressure at the translation level. Only three amino acid substitutions, located in the C-terminus, were detected. Linear B-cell epitope mapping predicted two epitopes in the Sal-1 PvETRAMP11.2 protein, one of which was fully conserved in all of the parasite populations analyzed.

**Conclusions:**

We provide an overview of the allele distribution and genetic differentiation of ETRAMP11.2 antigen in *P. vivax* populations from different endemic areas of the world. The reduced polymorphism and the high degree of protein conservation supports the application of PvETRAMP11.2 protein as a reliable antigen for application in serological assays or vaccine design. Our findings provide useful information that can be used to inform future study designs.

**Graphical abstract:**

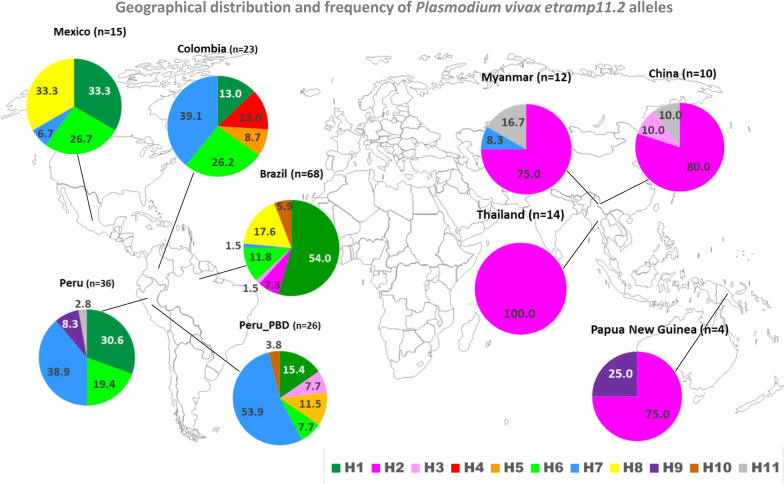

**Supplementary Information:**

The online version contains supplementary material available at 10.1186/s13071-023-05851-9.

## Background

*Plasmodium vivax* malaria is endemic in most of the world's malaria areas, with a global burden of 5.9–7.1 million estimated cases in 2020 [1]. Outside of Africa it accounts for 50% of all malaria cases, especially in the Middle East, Southern Asia and Western Pacific tropical regions where *P. vivax* parasites dominate. The control and elimination of* P. vivax* is particularly difficult due to several biological and epidemiological features, including the presence of hypnozoites, early production of mature gametocytes, low parasitemia and relapsing malaria. To achieve* P. vivax* elimination, new control strategies and vaccine development remain a priority. Currently, only one malaria vaccine, Mosquirix (RTS, S/AS01) (GSK plc, Brentford, UK), has reached the distribution stage [2]. This vaccine is based on the * Plasmodium falciparum* circumsporozoite surface protein (PfCSP) and is protective against* P. falciparum* malaria, albeit with a moderate efficacy. There are high expectations for the vaccine candidate R21/Matrix-M, currently in the approval process [3]. However, the prospects of an efficacious vaccine is worse for* P. vivax* malaria than for* P. falciparum* malaria. To date, there are only three antigens in the initial clinical phase (including *P. vivax* CSP [PvCSP]) and few candidates in preclinical trials [4]. Since the availability of a vaccine is likely to be long in coming, the control of *P. vivax* malaria needs to focus on appropriate surveillance systems.

Successful strategies for the control of *P. vivax* malaria require appropriate surveillance systems. Particular attention should be directed towards the assessment of transmission levels as this knowledge is crucial to understanding infection status and transmission dynamics. To this end, serological assays that measure malaria antibody responses are increasingly being used to estimate the malaria exposure of individuals in at-risk populations, with the aim to overcome the limitations of conventional diagnostic tests that are not sensitive enough to detect low-density malaria infections in malaria residual areas [5]. In these contexts, more sensitive serological tools [6] that are able to detect asymptomatic infections [7] and also suitable to improve epidemiological surveillance in low transmission settings are required.

Antibody responses to only a few parasite proteins have been specifically investigated to identify potential antigens for malaria serosurveillance, and most of these proteins are primarily target proteins of interest for malaria vaccine [8]. Unfortunately, most of the key immune targets exhibit a high degree of genetic polymorphism that may elicit allele-specific immune responses. To overcome the limitations of antigenic diversity, it is necessary to explore new serological markers and take into consideration their genetic variability [9], which might reduce the sensitivity of the assay [10]. Studying antigen polymorphisms in endemic populations would enable the selection of parasite proteins displaying limited variation [11] and therefore the most appropriate antigen variants to be used to improve serological assays for malaria surveillance or those to be included in effective vaccines.

One of the serological markers expressed in *P. vivax* is the early transcribed membrane protein (PvETRAMP) 11.2 (PVX_003565), a member of the PvETRAMP gene family that was first recognized in *P. falciparum* [12, 13]. Nine genes encoding proteins in the PvETRAMP family have recently been identified in *P. vivax* [14]. All of these genes have putative orthologs in *P. falciparum* [15].

PvETRAMP11.2 has been defined as antigenic target associated with immune response by immunogenic profiles of human sera against *P. vivax* proteins [16]. It has also been recognized by serum of malaria patients, and high antibody responses are elicited in mice immunized with the recombinant protein, suggesting a long-term immune response [15]. Among the members of the *P. vivax* ETRAMP family proteins, high serum positivity was found for PvETRAMP11.2 [14]. A blood stage proteome-wide microarray, used to screen the sera of *P. vivax*-exposed individuals, revealed PvETRAMP11.2 as one of the most abundant immunogenic proteins of *P. vivax* [17], strongly supporting its serodiagnostic potential. PvETRAMP11.2 was also suggested to be a potential vaccine candidate, similar to other members of the PvETRAMP family [14].

The PvETRAMP11.2 gene (*pvetramp11.2*) is a single-exon gene located on chromosome 4 which encodes a protein consisting of 110 amino acids (aa), with a predicted molecular weight of 12 kDa [14]. A signal peptide (aa positions 1–22) and two transmembrane domains (aa position 7–29 and 52–74, respectively) characterize the protein structure [15]. Transcriptome studies have shown that *pvetramp11.2* is one of the most expressed genes in *P. vivax* blood stage and schizont transcriptomes [18, 19]. The protein is associated with the parasitophorous vacuole membrane of blood stage parasites, but its function remains poorly understood [15].

Despite PvETRAMP11.2 being a well characterized antigen, its genetic variability has not been studied. Therefore, the aim of this study was to characterize the genetic variability of *pvetramp11.2* in *P. vivax* field samples of parasite populations collected from two endemic areas of the Brazilian and Peruvian Amazon. This study was conducted within the framework of a project supported by the European Commission project (EC_EraNet FP7 ELAC2015/T08-1061; acronym SEROVIVAX**),** aimed at developing inter-country standardized serosurveillance tools to accurately monitor changes in* P. vivax* transmission intensity in Amazonian regions. In addition, we analyzed allelic variants, using *pvetramp11.2* from publicly available sequences of clinical isolates from different malaria endemic regions worldwide. Linear B-cell epitopes were predicted and their polymorphisms analyzed. The present study reports the first analysis of *pvetramp11.2* diversity and provides baseline information to assess its usefulness as a potential antigen for implementing molecular surveillance tools and for malaria vaccine development.

## Methods

### Study area

The Amazon extends across nine countries in northern South America. Most malaria transmission occurs in riverine villages, farming settlements, gold mining camps and Amerindian reserves [20]. Amazonian Brazil and Peru together account for 25% of the current annual burden of malaria in the Americas [1].

### Samples

Samples of *P. vivax* populations from Brazil (*n* = 68) were collected in two sites in the state of Acre, northwestern Brazil. Two samples were collected during a cross-sectional survey conducted in rural sites around the town of Acrelândia in September–October 2004 [21], and 66 samples were obtained during a therapeutic efficacy study carried out in the town of Mâncio Lima between June 2014 and July 2015 [22]. Samples of Peruvian parasite populations (*n* = 36) were collected during epidemiological surveys conducted in Mazan district, in the northeastern Peruvian Amazon Department of Loreto between July 2015 and April 2017 [23, 24].

### DNA extraction

DNA was extracted from dried blood spot samples from the Peruvian collection using the Pure Link Genomic DNA Extraction Mini Kit (Invitrogen™, Thermo Fisher Scientific, Waltham, MA, USA) following the manufacturer's protocol. Purified DNA from Brazilian samples [21, 22] were stored at −20 °C until PCR analysis.

### PCR amplification and sequencing

Outer primers F1 (5ʹ-TGCTTGTCATCTCGAGTGGT-3ʹ) and R1 (5ʹ-TTTCCAACTCTGTTGCTTA-3ʹ) and internal primers F2 (5ʹ-GCTCAGGTCTTTGCTCCATG-3ʹ) and R2 (5ʹ- ACCTCTTCGCTGCTTTGTTAG-3ʹ) were designed based on the reference *pvetramp11.2* gene sequence of *P. vivax* Salvador-1 (Sal-1) strain (PVX_003565). The partial sequence of *pvetramp11.2* was amplified by nested (F2-R2) or semi-nested (F1-R2) PCR. All PCR reactions were performed using 0.5 U of GoTaq DNA Polymerase (Promega, Madison, WI, USA) in Green reaction buffer (Promega) plus 0.5 µl of 10 mM PCR Nucleotide Mix (Roche, Basel, Switzerland) and 0.1 µl of each primer pair (stock 100 pm/µl), in a final reaction volume of 25 µl containing 3 μl of template DNA. DNA amplifications were carried out with a pre-amplification denaturation step at 94 °C for 5 min, followed by 39 cycles of denaturation at 94 °C for 30 s, annealing at 55 °C for 30 s and extension at 72 °C for 1 min, with a final elongation at 72 °C for 5 min. All DNA amplifications were performed on a TurboCycler Lite thermo cycler (Blue-Ray Biotech Corp., New Taipei City, Taiwan). Amplicons were run in a 1.5% agarose gel in order to assess their amount, the correct size and absence of background or unspecific bands before sequencing. The PCR products were purified with the GeneAll Kit (GeneAll Biotechnology, Seoul, South Korea) following the manufacturer’s protocol, and then sequenced both in the forward and reverse directions by Eurofins Genomic. Sequencing primers were the upstream and downstream primers of the secondary PCR.

### Sequence data set

The sequence data set included in the present study comprises novel sequences from Brazilian (*n* = 68) and Peruvian parasite samples (*n* = 36) and additional sequences of *pvetramp11.2* (*n* = 104) from parasite samples collected in different geographical areas (Peru, Mexico, Colombia, China, Myanmar, Thailand and Papua New Guinea) that were downloaded from the PlasmoDB [25] database, with the overall aim to provide an overview of *pvetramp11.2* genetic polymorphisms in populations from different malaria endemic areas of the world (Additional file 1: Text S1).

The*pvetramp11.2* sequence of the curated reference strain PvP01 (PVP01_0422600) was used as the query to retrieve the FASTA files from the plasmoDB database, in the section “genetic variation.” Only *pvetramp11.2* sequences with no ambiguity in terms of nucleotides or gaps were selected, including from Peru (*n* = 26), China (*n* = 10), Mexico (*n* = 15), Papua New Guinea (*n* = 4), Colombia (*n* = 23), Thailand (*n* = 14) and Myanmar (*n* = 12). The Peruvian sequences retrieved from PlasmoDB were kept separate from those of the Peruvian population sequenced in the present study and named Peru_PDB. The *P. vivax* Salvador-1 *etramp11.2* sequence (Sal-1; PVX_003565) was used as reference for the alignments. A total data set of 208 sequences was generated. Nucleotide sequences were aligned using the ClustalW multiple sequence aligner in Accelrys Gene v2.5 (Accelrys Inc., BIOVIA, San Diego, CA, USA) and CLC-Main workbench v21 software (Qiagen, Hilden, Germany).

### Population genetic analysis

Statistical analyses were carried out using DnaSP v6 [26], DnaSP v5 [27] and GenAlEx v6.5. software [28, 29]. The number of alleles (H), segregating sites (Ss), allele diversity (Hd), singletons (Si), nucleotide diversity (Pi), average number of nucleotide differences (K), synonymous (Syn) and non-synonymous (N-Syn) mutations were calculated to infer population diversity.

Sliding window analysis of nucleotide diversity (Pi), with a window size of 10 bp and step size of 5 bp, was performed for all the populations (Papua New Guinea represented by only 4 samples and Thailand with only 1 allele were excluded from the analysis). Tajima’s D, Fu and Li’s D* and F* test statistics, carried out using DnaSP v5, were used to assess the selective pressure on the gene.

Pairwise genetic differentiation and its significance was estimated for all populations by the Wright fixation index (Fst) using Arlequin version 3.5 [30]. We set the number of permutations to 110 and the *P*-value significance level to *P* < 0.05.

### Protein analysis

The sequences from the total data set (*n* = 208) were translated into amino acid sequences applying the standard genetic code with DnaSP v6. Deduced proteins from reference sequences Brazil I, India VII, Mauritania, Chesson, Belem, IQ07 and PVP01 and field samples from parasite populations collected in Madagascar (M08, M15, M19) and Cambodia (C08, C15, C127) retrieved from the PlasmoDB database were added to the analysis.

### Prediction of linear B-cell epitopes

BepiPred 2.0 [31] and ABCpred [32] web-servers for sequence-based B-cell epitope prediction were used to investigate the presence of potential epitopes on the entire sequence of PvETRAMP11.2 (Sal-1; PVX_003565). A threshold of 0.50 (default value), which corresponds to the expected sensitivity and specificity of 0.59 and 0.57, respectively, was set for BepiPred, and the same analysis was performed with a threshold setting of 0.57. The antigenicity of the BepiPred-predicted epitopes was evaluated using the VaxiJen 2.0 server [33] with a default threshold of 0.5 for parasite mode. A threshold of 0.8 and window length of B-cell epitopes of 20 aa residues were applied in ABCpred, a server for alignment-independent prediction of protective antigens based on the physicochemical properties of amino acids sequences.

### Codon usage analysis

The extent of codon preference was estimated by the effective number of codons value (ENC), the measure that quantifies the effective number of codons used in a gene [34], and the Codon bias index (CBI), the measure of the extent to which preferred codons are used in a gene [35], using DnaSP v6 software. ENC values range from 20 to 61 (from maximum codon bias to no codon preference). CBI values range from 1 (maximum codon bias) and 0 (uniform use of synonymous codons). An ENC value < 40 is commonly treated as evidence of a strong codon usage bias [36].

The Fast Unconstrained Bayesian AppRoximation (FUBAR) method [37] implemented in the Data Monkey Web Server was applied to identify codons targeted by selection, setting a posterior probability of 0.9 (default value) for significant levels of codon deviation from neutrality.

## Results

### Nucleotide polymorphism and genetic diversity in Brazilian and Peruvian *P. vivax* parasite populations

**Fig. 1:**
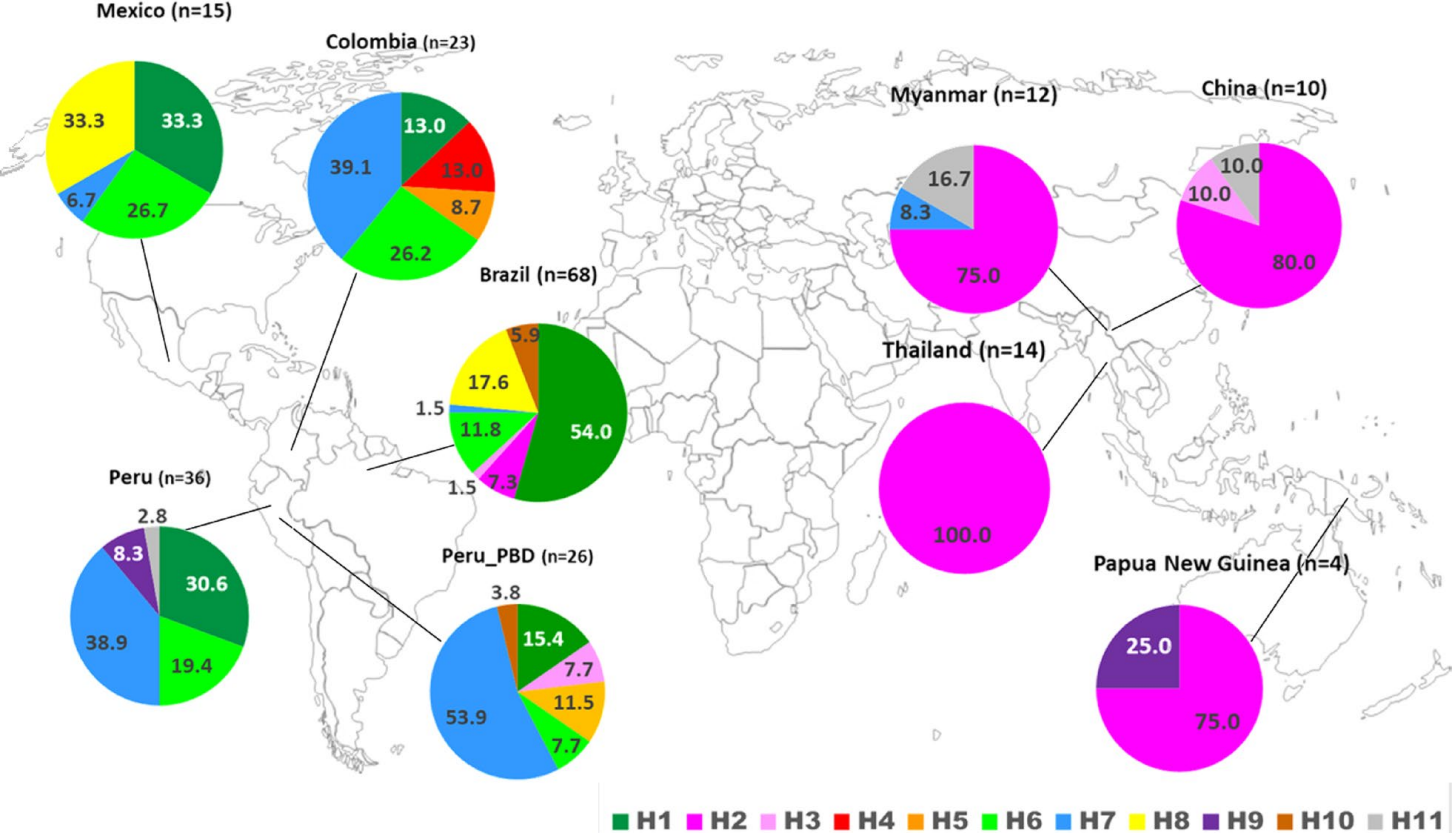
Geographical distribution and frequency of the alleles of *Plasmodium vivax* *etramp11.2* gene. Pie charts indicate the percentage of individual *pvetramp* alleles for each population and are mapped to their geographical location. The total number of field isolates from each geographical region is shown in parentheses. Colors defining the 1 alleles are given under the map

The partial sequence of 279 bp of *pvetramp11.2* (PVX_003565), corresponding to the region from nucleotides (nt) 55 to 333 of the Sal-1 reference sequence and encoding 92 aa residues (from aa 19 to 110) was successfully sequenced in 68 field samples from Brazil and 36 field samples from Peru. Brazilian samples showed one synonymous polymorphism (C198G) and four non-synonymous polymorphisms (A269G, G320T, A325G, T326G), giving rise to seven alleles (H1, H2, H3, H6, H7, H8, H10). The H1 allele (Sal-1 nucleotide sequence) was the predominant allele (54.4%) in the Brazilian parasite population. Peruvian samples showed four single nucleotide polymorphisms (SNPs) (C198G, A269G, G320T and T326G), which led to the identification of five different alleles (H1, H6, H7, H9 and H11). Peruvian and Brazilian isolates shared three alleles (H1, H6 and H7). In total, nine alleles were observed that showed marked geographical variation in terms of frequency (Table 1; Fig. 1).

**Table 1:** Nucleotide polymorphisms and allele frequencies of the *Plasmodium vivax* *etramp11.2* gene in natural isolates of *P. vivax*

Allele^a^	Nucleotide position							Latin America^b^					SEA/PNG^b^				
	66	96	198	269	320	325	326	Brazil	Peru	Peru_PDB	Colombia	Mexico	China	Myanmar	Papua NG	Thailand	Total
H1	C	A	C	A	G	A	T	37 (54.4)	11 (30.6)	4 (15.4)	3 (13.0)	5 (33.3)					60
H2	C	A	C	G	T	A	G	5(7.3)					8 (80.0)	9 (75.0)	3 (75.0)	14 (100.0)	39
H3	C	A	C	G	G	A	T	1 (1.5)		2 (7.7)			1 (10.0)				4
H4	C	G	C	G	T	A	G				3 (13.0)						3
H5	T	A	C	G	T	A	G			3 (11.5)	2 (8.7)						5
H6	C	A	G	A	T	A	G	8 (11.8)	7 (19.4)	2 (7.7)	6 (26.2)	4 (26.7)					27
H7	C	A	C	A	T	A	G	1 (1.5)	14 (38.9)	14 (53.9)	9 (39.1)	1 (6.7)		1 (8.3)			40
H8	C	A	G	A	G	A	T	12 (17.6)				5 (33.3)					17
H9	C	A	C	G	G	A	G		3 (8.3)						1 (25.0)		4
H10	C	A	C	G	T	G	G	4 (5.9)		1 (3.8)							5
H11	C	A	C	A	G	A	G		1 (2.8)				1 (10.0)	2 (16.7)			4
Total								68	36	26	23	15	10	12	4	14	208

*Pap NG* Papua New Guinea, *SEA/PNG* Southeast Asia/Papua New Guinea

^a^The 11 alleles are defined by the substitutions observed at 7 nucleotide positions. The nucleotides are numbered according to the Salvador-1 (Sal-1) reference strain, which corresponds to the H1 allele. Nucleotide substitutions are shown with underlining

^b^Values for frequencies are presented as the number of samples in which the allele was found, with the percentage given in parentheses

Minimal variation in the genetic diversity parameters was observed between parasites from the two countries (Brazil and Peru), and nucleotide diversity per site (Pi) values were also similar (0.00566 for Brazil and 0.00507 for Peru). The average number of nucleotide differences was marginally higher in parasites from Brazil (*K* = 1.579) than in those from Peru (K = 1.416). The allele diversity was higher in parasites from Peru (Hd = 0.730) than in those from Brazil (Hd = 0.659). A preponderance of non-synonymous over synonymous mutations was detected (Table 2).

**Table 2:** Estimates of genetic diversity statistics at* pvetramp11.2* among *P. vivax* populations

Population	Number of samples	Invariable(monomorphic) sites	Ss	H	Hd	K	Pi	Si (nt)	Syn (nt)	N-Syn (nt)
Brazil	68	274	5	7	0.65935	1.57858	0.00566	0	1 (198)	4 (269,320, 325, 326)
Peru	36	275	4	5	0.73016	1.41587	0.00507	0	1 (198)	3 (269, 320, 326)
Peru_PDB	26	273	6	6	0.68615	1.54462	0.00554	1 (325)	2 (66, 198)	4 (269,320,325,326)
Colombia	23	273	6	5	0.77075	1.63636	0.00587	0	3 (66, 96, 198)	3 (269, 320,326)
Mexico	15	276	3	4	0.75238	1.46667	0.00526	0	1 (198)	2 (320,326)
China	10	276	3	3	0.37800	0.75600	0.00271	2 (269,326)	0	3(269, 320, 326)
Myanmar	12	277	2	3	0.43939	0.71212	0.00255	0	0	2 (269, 320)
Thailand	14	279	0	1	0.00000	0.00000	0.00000	0	0	0
Papua NG	4	278	1	2	0.50000	0.50000	0.00179	1 (320)	0	1 (320)

*H* Number of alleles, *Hd* allele diversity, *K* average number of nucleotide differences, *N-Syn* non-synonymous changes, *nt* nucleotide position, *Pi* nucleotide diversity per site, *Si* number of singletons, *Ss* Number of segregating sites, *Syn* synonymous changes, Papua NG Papua New Guinea

### Genetic diversity of *P. vivax* etramp11.2 in different geographical regions

A panel of 208 sequences was used to evaluate the worldwide genetic variation of *pvetramp11.2*. The data set included *pvetramp11.2* sequences from Brazil (*n* = 68) and Peru (*n* = 36) generated in the present study, as well as additional *pvetramp11.2* sequences (*n* = 104) retrieved from the PlasmoDB database, including sequences from Peru_PDB (*n* = 26), Colombia (*n* = 23), Mexico (*n* = 15), Thailand (*n* = 14), China (*n* = 10), Papua New Guinea (*n* = 4) and Myanmar (*n* = 12). Analysis of the sequences retrieved from PlasmoDB confirmed the presence of all nine SNPs detected in the Brazilian and Peruvian samples evaluated in this study. Only two additional synonymous mutations were observed, C66T in samples from Colombia and Peru_PDB and A96G in samples from Colombia, which defined two additional alleles, H4 restricted to Colombia (13%) and H5 shared among Peru_PDB (11.5%) and Colombia (8.7%) (Table 1). The analysis of the total data set showed the presence of 11 alleles. Samples from the Latin American region included all alleles, while those from the SEA/PNG region included five alleles. The reduced allele diversity found in the SEA/PNG populations could also be a consequence of the low number of samples available for the analysis. Geographical differences in the allele distribution patterns were observed (Fig. 1). The H1, H6 and H7 alleles were shared by all Latin American populations, and the H2 allele predominated in SEA/PNG populations (Table 1). The H1 allele (Sal-1 allele) was the most highly represented allele in Brazil (54%) and was present at high frequencies in samples all other Latin American countries (range: 13.0–33.3%). H6 allele frequencies ranged from 7.7% in samples from Peru_PDB to 26.7% in samples from Mexico. The H7 allele was the most highly represented allele in Peruvian populations and those from Colombia (range: 38.9–53.9%), while it was poor represented in the samples from Brazil and Peru, the only two populations characterized by allele H8 (17.6% and 33.3%, respectively). The Sal-1 allele (H1) was not detected in any of SEA/PNG populations in which the dominant allele was H2; H2 was the only allele circulating in samples from Thailand and was poorly represented in those from Latin America (found in Brazil only: 7.3%). No allele was found to be fully shared by all populations.

*Pvetramp11.2* showed limited nucleotide diversity (Pi) among geographic locations (range: 0.00000–0.00587). The lowest levels of Pi were detected in SEA/PNG populations (range: 0.00000–0.00255) (Table 2). No synonymous mutation was detected in SEA/PNG populations. Allele diversity (Hd) and the average number of nucleotide differences (K) for individual populations ranged from 0.000 to 0.77075 and 0.0000 to 1.63636, respectively. The Pi of individual populations was calculated using the sliding window technique to detect regions of increased diversity. The sliding window plot showed values of heterogeneously distributed across the gene sequence and high values proximal to the 3' end of the coding region for all the populations (Additional file 2: Figure S1). Values in this region were above average levels calculated over the entire sequence.

### Population differentiation

To evaluate genetic differentiation among parasite populations, we estimated the fixation index (Fst). Significant inter-population differentiation was observed between the Latin American and the SEA/PNG parasite populations. Fst values also showed a statistically significant differentiation between populations from Brazil and Peru, as in most pairwise comparisons between Latin American populations. Low genetic differentiation was observed in parasite populations from the SEA/PNG area, suggesting the presence of gene flow among these populations. Results of this analysis are summarized in Table 3.

**Table 3:** Inter-population differentiation (fixation index) among *P. vivax* populations included in this study

Population	SEA/PNG				Latin America				
	Myanmar	China	Thailand	Papua NG	Colombia	Mexico	Peru_PDB	Brazil	Peru
Myanmar	–								
China	− 0.04792	–							
Thailand	0.16612	0.0978	–						
Papua NG	− 0.06667	− 0.1661	0.33597	–					
Colombia	0.18942*	0.2521*	0.39801*	0.25606*	–				
Mexico	0.57448*	0.5753*	0.74280*	0.57768*	0.30076*	–			
Peru_PDB	0.17257*	0.2279*	0.39487*	0.24280*	0.00527	0.29739*	–		
Brazil	0.46110*	0.4601*	0.58214*	0.47579*	0.31114*	0.03980	0.25433*	–	
Peru	0.31315*	0.3573*	0.51445*	0.37688*	0.06601*	0.17183*	0.02767	0.15123*	–

Values are the fixation index (Fst)

*Papua NG* Papua New Guinea

*Statistically significant at *P* ≤ 0.05

### Neutrality test for selection

Neutrality indices estimated by Tajima's D, Fu and Li’s D* and Fu and Li’s F* were calculated to detect deviation from the standard neutral model of evolution (Additional file 3: Table S1). No significant deviations from neutrality were detected. Although not statistically significant, Tajima's D, Fu and Li’s D* and Fu and Li’s F* for the parasite samples from Brazil, Peru and Mexico were > 1, which may suggest balancing selection or a decline in population size. The overall values of the neutrality tests in the parasite population samples from China were negative but not significant, providing slight evidence of an excess of low-frequency alleles resulting from population expansion or directional selection. A larger sample size would be needed to provide a more and accurate parameter estimate.

### PvETRAMP11.2 protein polymorphism

Amino acid changes were deduced from nucleotide sequences of the total data set to provide insight into protein variability among the *P. vivax* samples collected worldwide (Additional file 4: Table S2). Comparative analysis showed three substitutions (R90K, S107I, I109S/G) along the partial protein sequences (Additional file 5: Figure S2). The substitution (I109G) was only found in the Brazilian and Peru_PDB populations. The predominance of the H2 allele in the SEA/PNG populations gave rise to the protein sequence harboring all the three amino acid changes in comparison with Sal-1. The Sal-1 PvETRAMP11.2 protein sequence was not observed in SEA/PNG populations. Based on this analysis, Latin American parasites had greater protein heterogeneity (Additional file 5: Figure S2), which is consistent with the greater overall genetic diversity that was observed.

*Plasmodium vivax* reference sequences, including Brazil I, Mauritania, India VII, Chesson, Belem, PvP01, IQ07 and field isolates from Cambodia (C08, C127 and C15) and Madagascar (M08, M15 and M19), were also compared. (Additional file 4: Table S2). An additional amino acid change, A35P, was exclusively observed in the India VII strain. This change was not considered further as it was not confirmed in any other sample.

The protein sequences of the three samples from Cambodia were identical to that predominant in the SEA/PNG parasite populations. Substitutions earlier observed in the other geographical areas analyzed were also identified in samples from Africa (Mauritania and Madagascar).

### B-cell epitopes prediction

The Sal-1 PvETRAMP11.2 protein sequence was used as input to identify regions recognized as linear B-cell epitopes by the BepiPred 2.0 web-server set to a default threshold of 0.50. The analysis predicted the occurrence of two epitope regions corresponding to residues 23F-50Q and 77 K-107S (Additional file 5: Figure S2), and these were evaluated for antigenicity using the VaxiJen 2.0 server. The antigenicity values were 0.5200 and 1.1793, respectively, for the two epitopes. These antigenicity scores, both above the threshold of 0.5, indicated that both peptides are suitable antigens. Polymorphic residues (aa 90 and 107) were detected only in the region predicted to contain residues participating in the second B-cell epitope. Using a stringent threshold (corresponding to an increased specificity), only the K residue at position 90 was retained in the output and predicted to be part of an epitope.

The ABCpred web-server revealed eight B-cell epitopes of 20 residues. Two of these, 23F-42D and 90 K-109I, had a high score (> 0.8) and overlapped (partially or completely) with those predicted by BepiPred.

### Codon-based tests

Low polymorphism in a gene could be a result of a bias in codon usage. To better characterize the low genetic variability of* pvetramp11.2*, we analyzed the relative usage of synonymous codons along the *pvetramp11.2* gene fragment by estimating differential codon usage indices (ENC and CBI) to assess preferential use of the synonymous codons. All *P. vivax* populations from Latin America showed lower ENC values (range: 32.489–33.935) compared to SEA/PNG populations (range: 36.611–38.104). The ENC values of all populations analyzed suggested a preferential use of synonymous codons. CBI values for all populations ranged from 0.556 to 0.584, which is far from random codon usage (CBI = 0) [34]. Values for individual populations are reported in Table 4.

**Table 4:** Indices of preferential use of synonymous codons in *P. vivax* populations

Population	Number of samples	ENC	CBI
Brazil	68	33.296	0.561
Peru	36	32.767	0.562
Colombia	23	33.852	0.559
Mexico	15	32.489	0.556
Peru_PDB	26	33.935	0.564
Thailand	14	38.104	0.581
Myanmar	12	36.611	0.578
China	10	37.497	0.580
Papua NG	4	37.845	0.584

*CBI* Codon bias index, *ENC* effective number of codons, *Papua NG* Papua New Guinea

We also used the Fast Unconstrained Bayesian AppRoximation (FUBAR), with a posterior probability of 0.9, to identify sites targeted by selection. In this analysis, we grouped populations of Latin America and those of SEA/PNG as two groups, and the results for these two geographical areas showed evidence of selection only in the Latin America group. Episodic negative/purifying selection was detected at three codons (codons 22, 32 and 66) and evidence of episodic positive/diversifying selection was found only at codon 90.

## Discussion

This study provides evidence for limited genetic polymorphism in the *P. vivax etramp11.2* gene in parasite populations from Brazil and Peru. A low protein variability in these populations was concluded, based on the observation of only three amino acid substitutions (K90R, S107I, I109S/G). Changes in amino acids that were present in the samples of Brazilian and Peruvian parasites were shared by parasites of other populations of Latin America (Mexico, Colombia) as well as by those outside of Latin America (Asia, Papua New Guinea and Africa) although the prevalence did differ by country. The amino acid substitutions were found to be located in the region spanning aa 90 to 110 in the C-terminus, presumed to be oriented facing the host cytosol [13] and therefore less susceptible to structural constrains.

Two of the three polymorphic residues (aa 90 and 107) fell in one of the two predicted linear B-cell epitopes (77 K-107S). Amino acid substitutions could alter the epitope and consequently antibody recognition, or the amino acid changes may not necessarily represent key polymorphisms contributing to immune evasion. Antigenic diversity may be more restricted than DNA sequence diversity, as has been observed for *P. falciparum* AMA-1 alleles [38]. The predicted epitope has a high score (1.1793), as assessed by the VaxiJen 2.0 tool, indicating a high antigenicity propensity. We also identified a second putative B-cell epitope (23F-50Q) with a VaxiJen antigenicity value above the threshold of 0.5 (0.5200) that is conserved across all samples of parasite populations included in this study. Conserved epitopes have recently become a focus of strong interest for application in the design of broad-spectrum vaccines. Novel vaccine approaches explore protective epitopes in conserved protein regions as alternative means to overcome antigenic diversity [39]. Considering that evaluation of a wider geographical range is needed to extend the finding on a global scale, if this conserved potential B-cell epitope elicits protective immunity, it could be considered a broadly protective epitope that transcends strains and warrants further immunological and functional exploration.

We estimated that the parasite populations analyzed in the present study share similar patterns of nucleotide diversity (Pi), and nucleotides with high diversity were found in the terminal coding region, regardless of their geographical origin, which is consistent with the signature of a constrained sequence. The absence of polymorphisms in specific protein regions in parasite populations from very distant geographical areas may reflect the existence of functional and/or structural constraints that severely limit PvETRAMP11.2 to accept alternative modifications [40].

We found differences both in the number of alleles and in the prevalence of individual alleles in the samples of parasite populations from the different endemic regions considered. Latin American populations harbor all alleles identified, and H1 (Sal-1 allele) is highly represented. SEA/PNG populations have restricted allele diversity, and the H2 allele dominates in this area. The Sal-1 *etramp11.2* allele (H1) was absent in parasite populations in the SEA/PNG area, suggesting that any vaccine studies based on this reference strain should be performed with caution in this specific area.

Fst estimates indicated substantial genetic differentiation between the SEA/PNG and Latin American populations, despite the low sequence polymorphism observed. Most of the parasite populations from Latin America are clearly differentiated from those on the same continent as well as from SEA/PNG populations, possibly due to factors such as local adaptation and/or limited gene flow or random genetic drift. Low and non-significant Fst values in parasite populations from the SEA/PNG area suggest much less differentiated populations with respect to those of Latin America, based on the presence of the few circulating alleles, of which one predominant allele (H2, frequency range: 75–100%) is shared by the populations. The low differentiation between populations and the low number of alleles could facilitate vaccine study for this area.

As observed for *pvetramp11.2,* a limited sequence diversity has also been detected in other genes, such as the gene for the *P. vivax* proteins P41 [41], PvP12 and PvP38 [42], PvRON4 in Colombian isolates [43] and PvRAP-1 and PvRAP-2 [44]. Unfortunately, inferences on natural selection operating on low polymorphic genes is a difficult task [45, 46]. Many constraints and the small length of the PvETRAMP11.2 protein (110 aa) may reduce the number of possible mutation and mask evolving sites [47, 48]. Neutrality tests for selection (Tajima’s D, Fu and Li’s D*and F*) do not detect significant deviations from neutral expectations. Neutrality test values > 1 in some Latin American populations and negative values found in the samples of parasite populations from China could suggest distinct demographic and/or selective pressure acting on different geographical regions. The use of additional samples better representing the natural diversity of the populations will further improve the analysis. A non-random usage of codons in parasite isolates, favoring preferred codons for individual amino acids, could imply a low diversity as a consequence. To estimate the usage of codons in our samples, we used the codon bias indices ENC and CBI. Notably, the ENC values were < 40 for all populations (ENC < 35 in Latin American populations) and well below the calculated mean (52.18) reported in a genome-wide study on *P. vivax* [49]. This result indicates that a possible preference in codon usage and a selective pressure at the translation level cannot be excluded. Bias in codon usage is generally associated to highly expressed genes [50] and is higher for shorter proteins [51] like PvETRAMP11.2.

Codon usage bias is determined by a balance between selection and mutational bias [52]. Since codon bias was observed in parasite populations, we explored selective forces acting on individual codons, and deviation from selective neutrality was measured by a codon-based approach with FUBAR method. Parasite populations from the Latin American area, but not from the SEA/PNG area, showed evidence of selection at individual codons. The results indicate that two opposing forces of natural selection may be operating on the protein. Episodic negative/purifying selection was detected at three sites (codons 22, 32 and 66). The negatively selected codons occurred in the conserved protein region and are synonymous codons. Evidence of episodic positive/diversifying selection was found at codon 90. The non-synonymous mutation K90R mapped to the B-cell epitope region (aa 77–107) in the variable C-terminus of the protein. This site, which is under positive selection, could play a role in the escape from host immunity.

Limitations of this study include the absence of the full-length sequences (54 nucleotides missing). The geographic range considered does not allow us to clarify whether the results can be extended to global* P. vivax* populations, and study should be expanded to provide a better view of the distribution of pvETRAMP11.2 polymorphisms. Also, the small sample size may not represent the full range of variation in parasite populations, possibly resulting in incorrect diversity estimates.

## Conclusions

In conclusion, the main finding of the current study is the reduced genetic variation of PvETRAMP11.2 antigen. Given that most vaccine antigens are characterized by regions of high polymorphism [4], such as PvCSP, PvAMA1, PvMSP1, the low polymorphism of PvETRAMP11.2 is an ideal feature for effective serological assays and vaccine approaches in multiple endemic regions. Previous studies have characterized PvETRAMP11.2 as a serological marker that is highly expressed in blood-stage parasites and as a highly immunogenic protein in *P. vivax* malaria patients. Consequently, the high degree of protein conservation identified in our study further supports PvETRAMP11.2 as a promising target antigen that deserves to be further studied in depth.

The results of this study are useful for improving our understanding of *pvetramp11.2* diversity, which has been poorly characterized so far, and for providing information facilitating the identification of sequence features that may aid the design of improved serological assays and vaccine formulations.

## Supplementary Information

**Additional file 1. **List of *P. vivax* samples retrieved from Plasmo DB with their IDs.

**Additional file 2: Figure S1.** Sliding window plot of global *P. vivax etramp11.2* nucleotide diversity (Pi).

**Additional file 3: Table S1.** Neutrality test estimates for *pvetramp11.2* in *P. vivax* populations.

**Additional file 4: Table S2.** Amino acid sequence polymorphisms in *P. vivax* ETRAMP11.2 protein worldwide.

**Additional file 5: Figure S2.** Sal-1 PvETRAMP11.2 sequence, predicted B-cell epitopes and protein sequence conservation.

## Data Availability

The data set supporting the conclusions of this article is available in the PlasmoDB repository (https://plasmodb.org/plasmo/app) and sequence IDs analyzed are listed in the Additional file 1.

## Abbreviations

PvETRAMP11.2: *Plasmodium vivax* early transcribed membrane protein 11.2
Sal-1: Salvador-1
SEA/PNG: Southeast Asia/Papua New Guinea
SNP: Single nucleotide polymorphism
