# Supplementary material for: Reduced polymorphism of Plasmodium vivax early transcribed membrane protein (PvETRAMP) 11.2
Source: Parasit Vectors. 2023 Jul 17;16:238. doi: 10.1186/s13071-023-05851-9 (PMC10353105; doi:10.1186/s13071-023-05851-9)

Additional file 2: Figure S1. Sliding window plot of global *P. vivax etramp* nucleotide diversity (Pi). A window length of 10bp and a step size of 5bp were used. The nucleotide diversity (Pi) of each population is plotted against the nucleotide position (x-axis). Nucleotide positions are those of the Sal I strain. Papua New Guinea, with only four samples, and Thailand with only one allele, were excluded from the analysis.

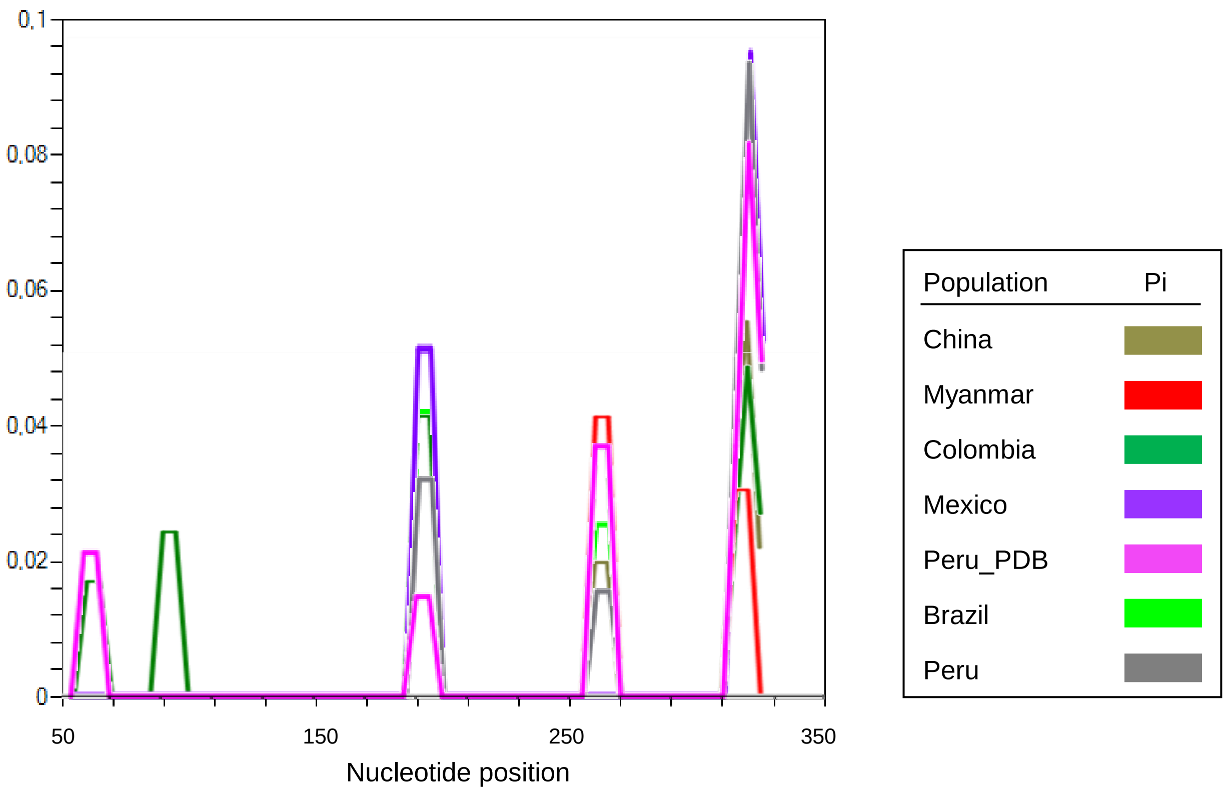

Supplement: Supplementary file 2 — Additional file 2: Figure S1. Sliding window plot of global P. vivax etramp11.2 nucleotide diversity (Pi). [file 13071_2023_5851_MOESM2_ESM.pdf]
