# Supplementary material for: Reduced polymorphism of Plasmodium vivax early transcribed membrane protein (PvETRAMP) 11.2
Source: Parasit Vectors. 2023 Jul 17;16:238. doi: 10.1186/s13071-023-05851-9 (PMC10353105; doi:10.1186/s13071-023-05851-9)
Supplement: Supplementary file 3 — Additional file 3: Table S1. Neutrality test estimates for pvetramp11.2 in P. vivax populations. [file 13071_2023_5851_MOESM3_ESM.docx]

| population | Tajima's D | Fu&Li D* | Fu&Li F* |
| --- | --- | --- | --- |
| Brazil | 1.16740 | 1.06977 | 1.29244 |
| Peru | 1.14592 | 1.03801 | 1.24499 |
| Peru_PDB | -0.05220 | 0.47619 | 0.37343 |
| Colombia | 0.02015 | 1.23613 | 1.02702 |
| Mexico | 1.75400 | 1.05657 | 1.41372 |
| China | -1.03446 | -0,80490 | -0.96179 |
| Myanmar | 0.21992 | 0.97295 | 0.88517 |
| Thailand | n.a. | n.a. | n.a. |
| Papua New Guinea | -0.61237 | -0.61237 | -0.47871 |

**Additional file 3: Table S1**. Neutrality test estimates for *Pv-etramp11.2* gene in *P. vivax* populations.

n.a., not applicable
