# Supplementary material for: Reduced polymorphism of Plasmodium vivax early transcribed membrane protein (PvETRAMP) 11.2
Source: Parasit Vectors. 2023 Jul 17;16:238. doi: 10.1186/s13071-023-05851-9 (PMC10353105; doi:10.1186/s13071-023-05851-9)
Supplement: Supplementary file 5 — Additional file 5: Figure S2. Sal-1 PvETRAMP11.2 sequence, predicted B-cell epitopes and protein sequence conservation. [file 13071_2023_5851_MOESM5_ESM.pptx]

## Slide 1
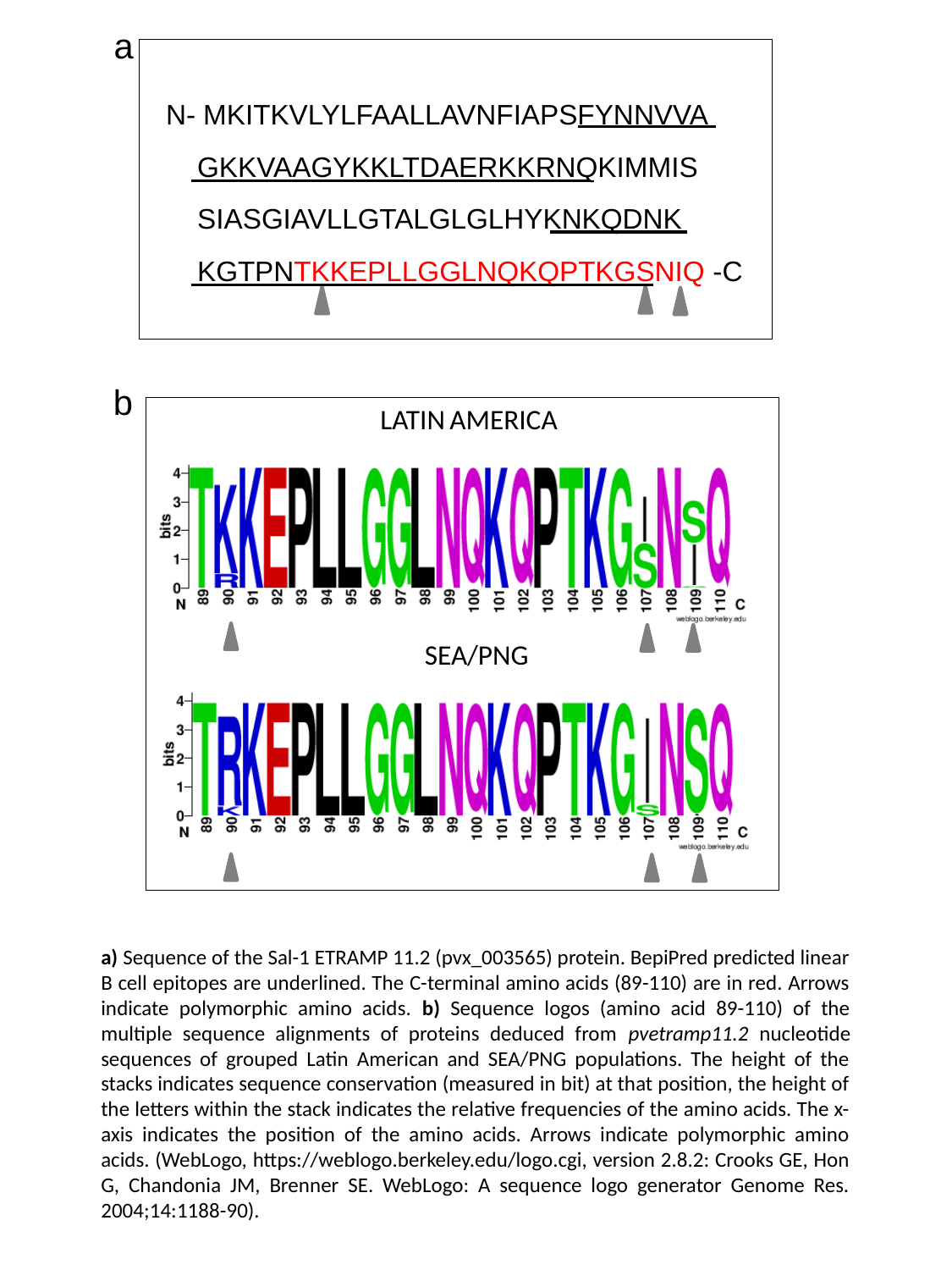

a
N- MKITKVLYLFAALLAVNFIAPSFYNNVVA
 GKKVAAGYKKLTDAERKKRNQKIMMIS
 SIASGIAVLLGTALGLGLHYKNKQDNK
 KGTPNTKKEPLLGGLNQKQPTKGSNIQ -C
b
LATIN AMERICA
SEA/PNG
a) Sequence of the Sal-1 ETRAMP 11.2 (pvx_003565) protein. BepiPred predicted linear B cell epitopes are underlined. The C-terminal amino acids (89-110) are in red. Arrows indicate polymorphic amino acids. b) Sequence logos (amino acid 89-110) of the multiple sequence alignments of proteins deduced from pvetramp11.2 nucleotide sequences of grouped Latin American and SEA/PNG populations. The height of the stacks indicates sequence conservation (measured in bit) at that position, the height of the letters within the stack indicates the relative frequencies of the amino acids. The x-axis indicates the position of the amino acids. Arrows indicate polymorphic amino acids. (WebLogo, https://weblogo.berkeley.edu/logo.cgi, version 2.8.2: Crooks GE, Hon G, Chandonia JM, Brenner SE. WebLogo: A sequence logo generator Genome Res. 2004;14:1188-90).
